# Coherent oscillations of a levitated birefringent microsphere in vacuum driven by nonconservative rotation-translation coupling

**DOI:** 10.1126/sciadv.aaz9858

**Published:** 2020-06-03

**Authors:** Yoshihiko Arita, Stephen H. Simpson, Pavel Zemánek, Kishan Dholakia

**Affiliations:** 1 SUPA, School of Physics & Astronomy, University of St Andrews, North Haugh, St Andrews KY16 9SS, UK.; 2 Molecular Chirality Research Centre, Chiba University, 1-33 Yayoi-cho, Inage-ku, Chiba-shi 263-0022, Japan.; 3 Institute of Scientific Instruments of the Czech Academy of Science, v.v.i., Královopolská 147, 612 64 Brno, Czech Republic.; 4 Graduate School of Science and Engineering, Chiba University, 1-33 Yayoi-cho, Inage-ku, Chiba-shi 263-0022, Japan.; 5 College of Optical Sciences, University of Arizona, Tucson, AZ 85721-0094, USA.; 6 Department of Physics, College of Science, Yonsei University, Seoul 03722, South Korea.

## Abstract

We demonstrate an effect whereby stochastic, thermal fluctuations combine with nonconservative optical forces to break detailed balance and produce increasingly coherent, apparently deterministic motion for a vacuum-trapped particle. The particle is birefringent and held in a linearly polarized Gaussian optical trap. It undergoes oscillations that grow rapidly in amplitude as the air pressure is reduced, seemingly in contradiction to the equipartition of energy. This behavior is reproduced in direct simulations and captured in a simplified analytical model, showing that the underlying mechanism involves nonsymmetric coupling between rotational and translational degrees of freedom. When parametrically driven, these self-sustained oscillators exhibit an ultranarrow linewidth of 2.2 μHz and an ultrahigh mechanical quality factor in excess of 2 × 10^8^ at room temperature. Last, nonequilibrium motion is seen to be a generic feature of optical vacuum traps, arising for any system with symmetry lower than that of a perfect isotropic microsphere in a Gaussian trap.

## Teaser

Coupling between the rotational and translational motions of an optically levitated microsphere leads to coherent oscillations.

## INTRODUCTION

The generation of coherent, directed motion in the mesoscopic regime is an issue of fundamental interest in the physical and biological sciences (*1*, *2*). On these length scales, energy fluctuations are comparable in size to the energy flows driving the system. At equilibrium, the principle of detailed balance holds: Any elementary process is equilibrated by its reverse process. Nevertheless, nature has devised a variety of schemes for overcoming this hurdle and extracting useful work from macromolecular machines, for example, (*3*). Among the numerous artificial analogs, colloidal heat engines are, perhaps, the simplest (*1*). Directed motion is extracted either by imposing temperature gradients or isothermally, through a ratcheting mechanism (*1*, *4*). In the latter case, time-varying potentials can give the required effect (*5*). Less commonly, a time-invariant, nonconservative force field is sufficient to push the system beyond equilibrium, breaking time inversion symmetry. Optical force fields, which are intrinsically nonconservative (*6*), fulfil this role. In optical tweezers operating at low Reynolds number, the effect is subtle, a delicate biasing of Brownian motion (*7*–*10*). As described here, the impact on underdamped systems is much more profound and can give rise to highly coherent oscillations that, when parametrically modulated, have quality factors in excess of 2 × 10^8^.

This paper reports an intriguing experimental observation: When an optically birefringent microsphere is held in a simple, linearly polarized Gaussian optical trap in vacuum, spontaneous oscillations emerge that grow rapidly in amplitude and become increasingly coherent as the air pressure, and, hence, the ambient viscosity, is reduced. This behavior diverges notably from the conventional understanding of optical tweezers, in which the equipartition of energy determines the position variance, 〈*x*^2^〉, of optically trapped (isotropic) spheres (*11*, *12*). In this case, the average potential energy of the particle in a trap with stiffness *K*, i.e., $\frac{1}{2}K\langle x^{2}\rangle$, is the same as the thermal energy, $\frac{1}{2}k_{B}T$. Equating these quantities shows that the position variance is completely independent of viscosity

$$\langle x^{2}\rangle =\frac{k_{B}T}{K}$$ (1)

This is an expression of the equipartition theorem and can be generalized to include multiple translational and rotational dimensions. Providing that the forces and torques can be derived from a scalar potential, the covariance of the coordinates will always be independent of viscosity since this quantity does not influence the potential (*13*). Any deviation from this behavior, including the effect described here, indicates a departure from equilibrium and equipartition.

Here, we describe the experiment in detail and show how these coherent oscillations also appear in direct numerical simulations. These simulations reveal the causative mechanism. The birefringence of the vaterite particle introduces nonsymmetric coupling between rotational and translational degrees of freedom. As a consequence, the optical forces surrounding the trapping point are linearly nonconservative: A repeated sequence of small translations and rotations, which return the particle to its initial configuration, result in a transfer of energy between the optical field and the particle. When excited by thermal forces, some trajectories grow in energy, accumulate momentum, and become increasingly coherent. It is these trajectories that we observe. In essence, the observed translational oscillations are driven by rotational oscillations and vice versa so that the combined oscillation becomes increasingly self-sustaining despite arising from stochastic thermal forces. This is a nonequilibrium steady state in which energy is continuously passed from the optical field to the particle and subsequently dissipated into the surrounding gas. Hence, the motion of the particle breaks time inversion symmetry. These features are captured by a simple analytical model.

Last, we show that by applying a parametric drive, we can reach extremely high values of the mechanical quality factor, *Q* ⪆ 2 × 10^8^, with ultranarrow linewidths of Δ*f* ≈ 2.2 μHz. We note that the concept of mechanical quality is typically associated with a harmonic oscillator in equilibrium with a thermal bath, which differs from the unconventional oscillator that we study here. Our oscillators are far from equilibrium and rely on the generation of highly coherent, stable, periodic motion. We may draw a parallel with the case for a levitated nanorotor torque sensor (*14*, *15*). An external force (or torque) results in measurable disturbances to the pure underlying signal. This form of measurement does differ from that associated with equilibrium devices but, importantly, does lead to exquisite sensitivity to environmental perturbations, as seen here for the specific case of varying gas pressure. The *Q* factors and linewidths that we report here have the most extreme values observed to date for these parameters, confirming the advantages of levitated microparticles for optomechanical studies. For comparison, a linewidth of 80 μHz was very recently reported for nanoparticles in a Paul trap with an associated quality factor of 1.5 × 10^6^ (*16*). While *Q* values as high as ≈10^12^ have been predicted for levitated nanoparticles in high vacuum (*17*), their measurement over appropriately long time periods is mitigated by thermal nonlinearities (*18*). Although the mechanisms responsible for the phenomena that we report (i.e., locally nonconservative optical forces) differ fundamentally from these cases, we note that their limits are yet to be explored and do not preclude high-precision measurements. By refining experimental parameters and investigating the physical limits on *Q* for these unconventional systems, further applications are sure to emerge.

## RESULTS

### Experiment

**Fig. 1:**
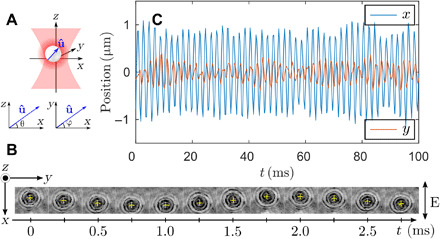
Experimental coordinates and oscillatory motion of a vaterite microsphere at the gas pressure of 1.2 mbar. (**A**) Trapping geometry and coordinate axes used throughout. The beam is polarized in the *x* direction and propagates in the positive *z* direction. The symmetry axis of the vaterite particle, $\hat{u}$, is specified in terms of the polar and azimuthal angles, θ and φ. (**B**) Time-lapse images of a vaterite microparticle oscillating along the **E**-field (*x* direction), where yellow crosses indicate the CoM of the particle. (**C**) Time traces of the particle’s CoM position both in *x* and *y* directions (transverse to the beam axis in the *z* direction).

Figure 1A shows the trapping geometry and coordinate axes used throughout this article. Vaterite microspheres have a spherulitic structure in which the optic axis of this highly birefringent material traces out nested hyperbolae arranged around a unique symmetry axis (section S1). A single vaterite microsphere with a radius of 2.2 μm is held in a Gaussian trap, linearly polarized in the *x* direction and propagating in the positive *z* direction with a wavelength of 1070 nm and beam power of 10 mW (measured at the back aperture of the microscope objective). Its motion is tracked with a fast complementary metal-oxide semiconductor (CMOS) camera (see Materials and Methods).

Figure 1B shows time-lapse images of the particle trapped at a residual gas pressure of 1.2 mbar [see movie S1, which is rendered at 15 frames per second (fps) from 5000 fps]. First, the particle aligns its symmetry axis ($\hat{u}$ in Fig. 1A) with the electric polarization (i.e., the *x* direction). The center of mass (CoM; yellow crosses) preferentially oscillates along the polarization direction with an amplitude that can be as large as ±1 μm compared to that of ±0.1 μm in the orthogonal *y* direction (Fig. 1C).

**Fig. 2:**
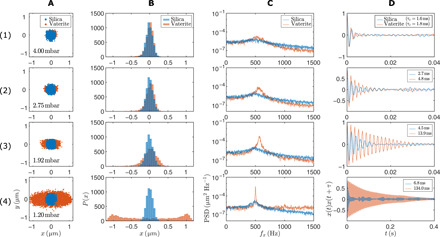
Experimentally measured dynamics of a vaterite microsphere compared with a silica microsphere trapped with a linearly polarized beam. (**A**) CoM position distributions in the *x*-*y* plane (transverse to the beam axis, *z*) and (**B**) their histograms in the *x* direction (along **E**-field). (**C**) Power spectra of *x*(*t*) showing the trap frequency at **∼**530 Hz. (**D**) Autocorrelation of *x*(*t*) normalized by 〈*x*^2^〉, where τ*_c_* denotes the correlation time. Rows (1) to (4) represent data at different gas pressures.

Figure 2 describes the strong dependence of the oscillatory *x* motion on the gas pressure. Column (A) shows the CoM positions of the vaterite particle in the *x*-*y* plane (red dots) measured at every 0.2 ms for the duration of 1 s (5000 data points in total) at different gas pressures. The oscillation amplitude in the *x* direction increases as the residual gas pressure is reduced by a few millibars, from 4 to 1.2 mbar. The motion is compared with that of an isotropic silica microsphere with a radius of 2.5 μm under the same trapping conditions (Fig. 2A, blue dots), confirming that the oscillatory motion is unique to the birefringent particle. Distributions of the *x* coordinate are given for vaterite and silica particles in Fig. 2B. In accordance with the equipartition of energy (Eq. 1), the distributions for silica are Gaussian and approximately independent of pressure, while those for vaterite broaden with decreasing pressure, finally changing form altogether. The power spectrum of the *x* coordinate clearly indicates the trap or oscillation frequency at around *f_x_* (=ω*_x_*/2π) ∼ 530 Hz, which remains the same at lower pressures (see red curves in Fig. 2C). Notably, the trapped vaterite particle exhibits much larger *Q* factors than those of the silica particle (blue curves), and kinetic energy is increasingly concentrated at a single resonant frequency.

Autocorrelation 〈*x*(*t*)*x*(*t* + τ)〉 (Fig. 2D) measures the rate at which the translational *x*(*t*) motion loses coherence because of thermal fluctuations. Vaterite exhibits an order of magnitude longer decay time than that of the silica (Fig. 2D, 4). These features correspond to greatly increased *Q* values for vaterite that, as discussed below, can be further enhanced through parametric driving. We note that as the gas pressure is decreased, inertial forces tend to exceed gradient forces, and the particle may leave the trap typically at a residual gas pressure of <1 mbar, depending on the optical power and the mass of the particle.

**Fig. 3:**
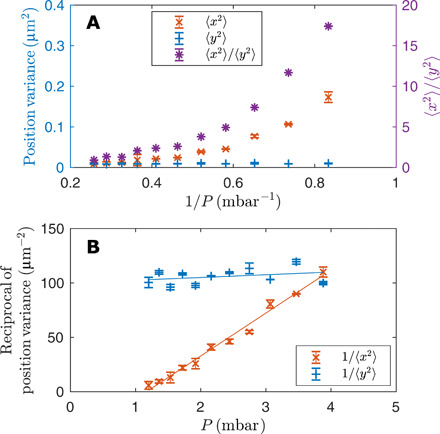
Experimentally measured position variance and its reciprocal of a vaterite microsphere. (**A**) Position variance in both *x* and *y* directions (left axis) and the relative energy in the *x* motion (right axis) at different gas pressures *P*. 〈*x*^2^〉 increases with decreasing *P* toward 1 mbar, whereas 〈*y*^2^〉 remains at room temperature. (**B**) The same data as (A), replotted as the reciprocal of the variance versus pressure to show the scaling behavior as the critical viscosity and pressure are approached.

Last, Fig. 3A shows the position variance of the trapped particle in the *x* direction (red crosses) compared with that in the *y* direction (blue plus signs), where 〈*y*^2^〉 is directly associated with the thermal energy through $\frac{1}{2}k_{B}T=\frac{1}{2}K_{\mathrm{yy}}\langle y^{2}\rangle$, where *K_yy_* is the trap stiffness in the *y* direction and 〈*y*^2^〉 = 4.49 × 10^−3^ μm^2^ at the pressure *P* = 4 mbar, assuming that the particle is at room temperature. The ratio 〈*x*^2^〉/〈*y*^2^〉 therefore approximates the relative energy in the *x* motion (Fig. 3A on the right axis). Figure 3B shows the reciprocal of the position variance against *P*, where experimental data are fitted with linear regression. Here, we clearly show the linear relationship of 1/〈*x*^2^〉 with *P*, which is predicted by the theory (see Eq. 13).

### Simulation

To better understand the experimental observations, we performed direct numerical simulations of the thermal motion of a homogeneous birefringent sphere with default parameters (radius of 2.2 μm and density ρ = 2.54 g/cm^3^ with ordinary and extraordinary refractive indices corresponding to those of bulk vaterite *n_o_* = 1.55, *n_e_* = 1.65) in a linearly polarized Gaussian beam in vacuum (see section S3 for further details). In summary, we numerically integrate the equation of motion

$$f^{\text{opt}}(q)+f^{L}(t)-\mathrm{mg}\hat{z}-\Xi \dot{q}=M\ddot{q}$$ (2)

**f**^opt^is the generalized optical force (i.e., forces and torques) acting on the particle, and **q** are the generalized coordinates specifying the CoM and orientation [i.e., **q** = (*x*, *y*, *z*, θ, ϕ), where θ and ϕ are the polar and azimuthal angles specifying the symmetry axis of the particle, $\hat{u}$]. **Ξ** is the pressure-dependent friction matrix with diagonal elements Ξ*_ii_* = 6πμ*a* and 8πμ*a*^3^ for translational and rotational motion, respectively. **f**^L^ is the stochastic, Langevin force (and torque), uncorrelated, with zero mean and amplitude fixed by the fluctuation-dissipation theorem, i.e., $\langle f_{i}^{L}(t)\rangle =0$, $\langle f_{i}^{L}(t)f_{j}^{L}(t\prime )\rangle =2k_{B}T\Xi_{\mathrm{ij}}\delta (t-t\prime )$. **M** is a diagonal matrix whose elements are given by the mass (*m*) of the sphere and its moment of inertia (*I*), and $-\mathrm{mg}\hat{z}$ is the weight. The beam propagates in the positive *z* direction with its axis coincident with the *z* axis. Although the model is idealized (ignoring the inhomogeneity and roughness of the particle, aberrations in the optical beam, etc.), the behavior represented in Fig. 2 is convincingly reproduced (see fig. S3). In particular, fig. S3 shows simulated distributions of the CoM, power spectra, and autocorrelation functions, which should be compared with Fig. 2 (A, C, and D, respectively).

**Fig. 4:**
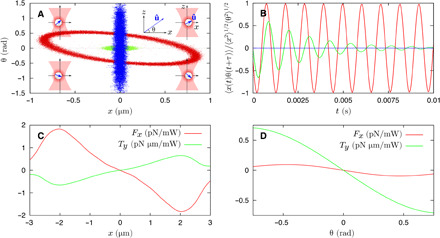
Simulations describing the correlation between the *x* and θ coordinates and their coupling forces and torques. (**A** and **B**) The correlation between *x* and θ for three separate cases: a birefringent sphere (Δ*n* = 0.1) at low air viscosity (μ = 1 × 10^−6^ Pa s) (red), the same birefringent sphere at a higher viscosity (μ = 3.25 × 10^−6^ Pa s) (green), and an isotropic sphere (Δ*n* = 0) at low viscosity (μ = 1 × 10^−6^ Pa s) (blue). (A) Scatter plot of the simulated coordinates. The insets at each corner show a complete cycle of successive rotations about the *y* axis and translations along the *x* axis (i.e., the electric polarization direction). (B) Cross-correlations, 〈*x*(*t*)θ(*t* + τ)〉, evaluated from the simulation data. (**C** and **D**) Calculations of the coupled forces *F_x_* and torques *T_y_* for *x* displacements (with θ = 0) and θ rotations (with *x* = 0), respectively.

In addition, the simulations reveal strong coupling between the rotational and translational degrees of freedom of the sphere. As the viscosity is reduced, the growing oscillation of the *x* coordinate of the CoM is increasingly accompanied by an oscillation of the angular coordinate, θ. Figure 4 shows this correlation as a scatterplot (left-hand side) and as the time-dependent cross-correlation of a simulated Brownian trail. The underlying mechanism is clear. Angular oscillations produce oscillating force components, $f_{x}^{\text{opt}}$, through rotation-translation coupling, which drive linear oscillations in *x*. Similarly, oscillations in *x* generate torque components, $t_{y}^{\text{opt}}$, which feed into the angular oscillations. As the pressure (viscosity) is reduced, the coupled oscillations increasingly reinforce one another and the motion becomes more coherent and self-sustaining. This effect is very clearly seen in movies S2 and S3, which show the simulated stochastic motion in optical traps whose parameters correspond to the three cases represented in Fig. 4 (A and B).

### Analysis

These observations can be quantified with reference to a simple analytical model. Linearizing the force field relative to the trapping configuration (in which external forces and torques vanish) and restricting attention to the coupled coordinates, θ and *x*, give

$$-Kq-\Xi \dot{q}+f^{L}=M\ddot{q}⟺(K+i\omega \Xi -M\omega^{2})Q\equiv AQ=F^{L}$$ (3)

The first expression is the linearized equation of motion. The external (i.e., optical and gravitational) forces on the particle are given by $f^{\text{opt}}-\mathrm{mg}\hat{z}\approx -Kq$, where $K=-{\left[\nabla (f^{\text{opt}}-\mathrm{mg}\hat{z})\right]}^{T}$ is a stiffness matrix that includes coupling coefficients e.g.

$$K=[\begin{array}{cc} K_{\mathrm{xx}} & K_{x\theta }\\ K_{\theta x} & K_{\theta \theta } \end{array}]$$ (4)

and **q** = (*x*, θ) are the coupled coordinates. **Ξ** is the friction matrix for a sphere of radius *a* in a fluid with viscosity μ, with diagonal components ξ*_xx_* = 6πμ*a* for translation and ξ_θθ_ = 8πμ*a*^3^ for rotation. The second expression in Eq. 3 is the frequency domain equivalent, with **Q** = (*X*, Θ), the Fourier transform of **q**. The eigenfrequencies of **A**, denoted by ω*_i_*, determine qualitative features of the trap including its linear stability, power spectral density (PSD), and correlation functions. In particular, if **K** is symmetric, then the force is locally conservative with (∇ × **f**) = 0 and the trap is at thermal equilibrium. However, this need not be the case for optical fields or other momentum flows (*8*). Complete calculations of these quantities are provided in the Supplementary Materials, and the main results are quoted and discussed below.

The real parts of the eigenfrequencies, $R(\omega_{i})$, describe oscillatory behavior. The imaginary parts, $I(\omega_{i})$, relate to relaxation [when $I(\omega_{i})>0$] or linear instability $[I(\omega_{i})<0$]. For isotropic spheres, coupling is absent and *K*_*x*θ_ = *K*_θ*x*_ = 0. The eigenfrequencies are then

$$\omega_{i}\approx \pm \sqrt{K_{\mathrm{xx}}/m}+i\xi_{\mathrm{xx}}/2m$$ (5)

so that the sphere oscillates with the natural frequency of a harmonic trap, while its relaxation is determined by the Stokes’ drag and the mass; the trap is linearly stable with $I(\omega_{i})=\xi_{\mathrm{xx}}/2m>0$. These traps are necessarily conservative within the linear regime and remain at equilibrium. When motional degrees of freedom are coupled, this constraint is lost. It is helpful to consider two extremes: the overdamped or low–Reynolds number regime (negligible inertia) and the underdamped limit (negligible viscosity).

In the low–Reynolds number regime, the eigenfrequencies are purely imaginary and positive unless *K*_*x*θ_*K*_θ*x*_ > *K_xx_K*_θθ_ (see section S4). This is a demanding condition, equivalent to the requirement that the coupling forces and torques exceed the restoring terms, and unlikely to be satisfied.

In the low-pressure limit, where viscosity is negligible, we have

$$\omega_{i}^{2}=\frac{1}{2}(\frac{K_{\theta \theta }}{I}+\frac{K_{\mathrm{xx}}}{m})\pm \sqrt{\Delta_{\text{LP}}}$$ (6)

with

$$\Delta_{\text{LP}}=\frac{1}{4}{\left(\frac{K_{\theta \theta }}{I}-\frac{K_{\mathrm{xx}}}{m}\right)}^{2}+\frac{K_{x\theta }K_{\theta x}}{\mathrm{mI}}$$ (7)

Setting the *K*_*x*θ_ = *K*_θ*x*_ = 0 results in the familiar natural frequencies for harmonic oscillators, e.g., $\omega_{i}^{2}=K_{\mathrm{xx}}/m,K_{\theta \theta }/I$. Characteristic frequencies with negative imaginary parts emerge whenever Δ_LP_ < 0, destabilizing the trap. This requires that *K*_*x*θ_ and *K*_θ*x*_ have opposite signs in Eq. 7. Physically, this condition ensures that the rotational and translational vibrations feed into one another constructively so that, for example, the rotational vibration generates an oscillating force that is in phase with the translational vibration and vice versa. Providing that *K*_*x*θ_*K*_θ*x*_ < 0, the condition Δ_LP_ < 0 is relatively easily satisfied, especially when the natural rotational and translational frequencies are close as they are in this case. Calculated forces and torques are shown in Fig. 4 (C and D) for the default parameters given above, showing that *K*_*x*θ_*K*_θ*x*_ < 0 as required. We note that calculated values of Δ_LP_ are negative for the default system parameters and for surrounding regions of parameter space (see section S4).

Whenever the trap is linearly stable at low Reynolds number (as usual), but unstable in the low-pressure limit (Δ_LP_ < 0 in Eq. 7), one of the values of $I(\omega_{i})$ must pass through zero as the ambient effective viscosity is reduced. Requiring $I(\omega_{i})=0$ yields the following expression for the critical effective viscosity, μ*_X_*, at which one of the $I(\omega_{i})=0$ (see section S4)

$$s_{x}s_{\theta }\mu_{X}^{2}=\mathrm{mI}\omega_{X}^{2}-(mK_{\theta \theta }+IK_{\mathrm{xx}})+(K_{\mathrm{xx}}K_{\theta \theta }-K_{x\theta }K_{\theta x})/\omega_{X}^{2}$$ (8)

where the friction coefficients ξ*_xx_* = 6πμ*a* ≡ *s_x_*μ and ξ_θθ_ = 8πμ*a*^3^ ≡ *s*_θ_μ are proportional to the viscosity and ω*_X_* is the corresponding real part of the frequency

$$\omega_{X}^{2}=\frac{K_{\mathrm{xx}}s_{\theta }+K_{\theta \theta }s_{x}}{ms_{\theta }+Is_{x}}$$ (9)

In the absence of rotational-translational coupling (*K*_*x*θ_ = *K*_θ*x*_ = 0), Eq. 8 gives μ*_X_* = 0, and when the condition $I(\omega_{i})=0$ cannot be satisfied (because *K*_*x*θ_*K*_θ*x*_ > 0, for example), a negative value is obtained for $\mu_{X}^{2}$, indicating a nonphysical solution. A survey describing typical variations in μ*_X_* and ω*_X_* with varying properties of the sphere and beam is included in section S4. The properties of the experimental system fall within realistic ranges given uncertainties in the beam power, for example, and the idealized nature of the model.

As μ → μ*_X_*, the oscillations become more coherent and grow in amplitude. This is expressed in the PSD, which describes the distribution of motional power in frequency space and is given by the ensemble average of the squares of the Fourier-transformed coordinates, 〈**Q**(ω)Q*(ω)〉. As $I(\omega_{i})$ approaches zero, the PSD for the vaterite sphere, with rotation-translation coupling, consists of a single peak with the maximum value, 〈**Q**(ω)Q*(ω)〉_max_, and width, Δω (section S4)

$${\langle Q(\omega )Q^{*}(\omega )\rangle }_{\text{max}}\propto \frac{1}{\omega_{X}^{2}I{\left(\omega_{i}\right)}^{2}}$$ (10)

$$\Delta \omega \propto 2I(\omega_{i})$$ (11)

Thus, the height of the peak in the PSD increases rapidly as μ*_X_* is approached and its width, Δω, decreases toward zero. Time correlations take the following form (section S4)

$$\langle q(t)q(t+\tau )\rangle \propto \frac{k_{B}T}{I(\omega_{i})}e^{iR(\omega_{i})\tau }e^{-I(\omega_{i})\tau }$$ (12)

As $I(\omega_{i})\to 0$, the amplitude $k_{B}T/I(\omega_{i})$ increases so that, for example, the instantaneous position variance, 〈*x*^2^〉, grows. Since the relationship between pressure and viscosity is approximately linear in this regime (*19*), we can write $I(\omega_{i})\propto (\mu -\mu_{X})\propto (P-P_{X})$, where *P_X_* is the pressure corresponding to μ*_X_*. Combining with Eqs. 10 and 12 gives the scaling behavior for the PSD maxima and linewidths as well as covariance and relaxation times. For example, 〈*x*^2^〉 is

$$\langle x^{2}\rangle \propto \frac{1}{P-P_{X}}$$ (13)

where *P_X_* is the pressure corresponding to the critical viscosity, μ*_X_*. In Fig. 3B, we confirm that this relation holds for the experimental data and use it to extract a value for *P_X_* = 1.2 mbar. Previous measurements (see section S2) show that the corresponding viscosity is μ*_X_* = 0.2 μPa s, which falls within the range predicted in the parametric survey (section S4). Similarly, the relaxation time, $\tau_{0}=1/I(\omega_{i})\propto 1/(P-P_{X})$ becomes large as μ → μ*_X_*, and the oscillations of the birefringent sphere become increasingly coherent.

The behavior described above, in Eqs. 10 to 12, is qualitatively different from that of isotropic spheres, for which the position variance, 〈*x*^2^〉, is independent of viscosity (Eq. 1), while the value of $I(\omega_{i})=\xi_{\mathrm{xx}}/2m$ remains finite for finite viscosity, ensuring that the linewidth and relaxation times also remain finite.

In summary, whenever Δ_LP_ < 0, the stochastic motion within the linear approximation is characterized by a critical viscosity, μ*_X_*. For μ > μ*_X_*, the motion is driven by thermal fluctuations, without which the particle would remain motionless in the trapping configuration. As the viscosity is reduced toward μ*_X_*, spontaneous oscillations emerge with a linewidth, Δω, approaching zero and quality factor $Q=\omega /\Delta \omega \approx \omega_{X}/I(\omega_{i})$ tending to infinity. In reality, this limit is never reached since the position variance, 〈*x*^2^〉, increases rapidly, taking the particle beyond the linear range of the trap. For viscosities μ < μ*_X_*, the linear approximation breaks down completely and the oscillations become self-sustaining. In essence, the particle takes energy from the optical field and dissipates it into the surrounding gas, forming a nonequilibrium steady state. The oscillations may be regarded as being driven, but the external force field is time invariant. Thermal fluctuations introduce phase errors that the system cannot correct since it has no knowledge of what the phase should be at any particular time. These errors accumulate and limit how narrow the linewidth can become.

### Ultrahigh *Q* oscillators

Although the extremely high *Q* values predicted by the linear approximation (Eq. 11) cannot be obtained in practice, parametric modulation of the trap intensity is observed to drastically improve the quality factors of the free-running oscillator. This remarkable effect is due, in part, to the oscillations being confined to a single spatial dimension, analogous to the case of the tethered pendulum (*20*). In addition, the parametric drive introduces a periodic time variation against which phase errors can be measured, allowing the oscillator to synchronize with the drive (*21*, *22*).

**Fig. 5:**
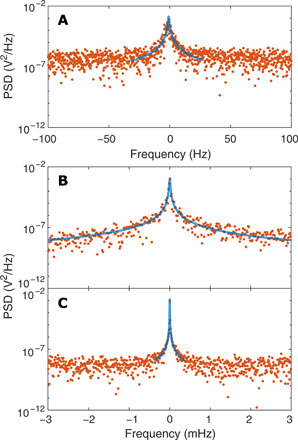
PSD with different oscillation modes. Experimentally measured PSD (orange dots) fitted with a Lorentzian (blue curves) of the oscillating particle at the oscillation frequency in the range from 530 to 840 Hz (centered at 0 Hz): (**A**) Self-sustained oscillations showing a linewidth Δ*f*_0_ of 0.90 Hz, yielding *Q* = 924. (**B**) Phase-locked oscillations showing Δ*f*_0_ = 10.5 μHz and *Q* = 5.53 × 10^7^. (**C**) Frequency-locked oscillations showing Δ*f*_0_ = 2.20 μHz and *Q* = 2.45 × 10^8^.

Figure 5A shows the PSD of the oscillating particle at around the resonant frequency *f*_0_ = 832 Hz (centered at 0 Hz). When the particle is trapped with a stationary light field both spatially and temporally, the oscillation yields the resonant peak (orange dots), which is fitted with a Lorentzian (blue curve) with a linewidth of 0.90 ± 0.13 Hz, yielding *Q* = 924 ± 136 (Fig. 5A). Because of collisions with residual gas molecules and CoM excursion of the particle into regions of different light intensity, the oscillation frequencies exhibit a broad distribution.

However, when the particle is trapped by a light field with an amplitude periodically modulated at a frequency *f* = 2*f*_0_ (i.e., parametrically driven), the mechanical mode becomes more coherent, leading to an ultranarrow linewidth. In this regime, there are two driving protocols: (i) The external modulation can be locked to the phase of the particle motion (either inward or outward, relative to the trap center), i.e., “phase locking.” (ii) The particle motion can be locked to the external clock, transducing the frequency of the time standard to the particle motion, i.e., “frequency locking” (see Materials and Methods). Figure 5B shows the PSD when phase locked, exhibiting a linewidth of 10.5 ± 3.0 μHz at *f*_0_ ≈ 580.5 Hz and the corresponding *Q* = 5.53 ( ± 1.72) × 10^7^. These mechanical properties are further improved when frequency locked. In this case, the particle acts as a precision micromechanical transducer, allowing long-time measurements required for high-*Q* experiments. Figure 5C shows the PSD with a linewidth of 2.20 ± 0.62 μHz at *f*_0_ ≈ 539.6 Hz and *Q* = 2.45 ( ± 0.75) × 10^8^ when frequency locked. These are, to the best of our knowledge, the narrowest linewidth and the highest *Q* factor reported to date for a mechanical oscillator at room temperature.

**Fig. 6:**
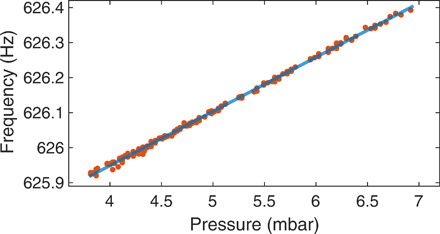
Pressure measurement by a phase-locked oscillator. The oscillation frequency (orange dots) fitted with a linear function (blue line) is monitored, while the pressure is changed in the range of 4 to 7 mbar.

To demonstrate weak-force sensing capabilities of the driven oscillators, the phase-locked particle is used as a probe for real-time frequency measurements in a dilute gas (see Materials and Methods). A fractional change in gas pressure affects the damping rate of the particle, which translates gas pressure values onto the oscillation frequency. Figure 6 shows the oscillation frequencies (orange dots) depending on the residual gas pressure in the pressure range of 4 to 7 mbar. A linear fit to the data (blue solid line) yields a rate of frequency change of 0.21 Hz/mbar with a relative pressure sensitivity of 0.75% (2σ), which can be improved by increasing the length of the measurement (currently 100 s). We note that frequency locking can also be used, where the phase lag between the drive and the oscillator depends on nonconservative forces, such as light or gas scattering (*15*). This demonstrates the great potential of this self-sustained oscillator as a weak-force sensor.

## DISCUSSION

In conclusion, we have demonstrated an effect in which thermal fluctuations combine with a linearly nonconservative optical force field to produce oscillations in one spatial direction whose amplitude and coherence grow rapidly as viscous damping is reduced. This is a nonequilibrium effect, and the motional energy greatly exceeds the thermal energy, $\frac{1}{2}k_{B}T$. The effect is loosely analogous to that previously reported for isotropic spheres in circularly polarized beams (*23*), except that, in this case, the nonconservative motion is induced by particle anisotropy rather than by momentum flows within the trapping beam. Since the optical forces experienced by a particle depend qualitatively on its shape (*24*–*27*), a range of exotic behaviors may be anticipated for particles of yet lower symmetry.

The phenomenon that we report belongs to a general class of nonconservative instabilities that manifest themselves in diverse physical systems ranging from biomechanics to magnetohydrodynamics (*28*). Structured optical fields and the complex force fields that they generate provide an ideal platform for studying these effects (*29*–*33*) and their thermodynamic consequences (*34*–*37*). Last, we have shown that the quality of oscillations generated by nonconservative coupling can be massively enhanced by parametric driving. The quality factors of our oscillating vaterite particles are the highest of those measured and exhibit the lowest linewidths seen to date. Nevertheless, we expect to be able to improve on these values by refining the size, shape, or composition of the particle or by using optimally structured light fields. In summary, our results expose the potentially rich behavior exhibited by optically trapped, nonspherical particles in vacuum optical traps and suggest unconventional forms of ultrahigh-quality oscillators for applications involving weak-force measurement.

## MATERIALS AND METHODS

### Sample preparation

The synthesis of vaterite microspheres and the protocol for loading the microspheres into the optical trap are described elsewhere (*29*). Vaterite is a positive uniaxial birefringent material in a spherical morphology with a mean radius of 2.20 ± 0.02 μm (2σ) and a surface roughness of 27.6 nm (2σ) (see fig. S1). As for silica, National Institute of Standards and Technology traceable size standards with a diameter of 5.1 ± 0.5 μm (Thermo Scientific, 9005) are used to compare the trapping behavior with vaterite microspheres (see Fig. 2).

### Experimental protocol

To load microspheres into the optical trap, we used an annular piezoelectric transducer (APC International Ltd., catalog no.70-2221) affixed to the vacuum chamber made of stainless steel with a volume of 27.7 μℓ. The chamber has two optical glass windows (Harvard Apparatus Ltd., CS-8R: 8 mm in diameter and 150 μm in thickness); upon the surface of one, we placed dried microspheres before loading. The piezoelectric transducer is operated at 140 kHz to detach microspheres from the glass surface to load the optical trap in air/vacuum. A linearly polarized trapping beam (continuous wave, 1070 nm) with optical power in the range of 10 to 25 mW is focused in the vacuum chamber through a high–numerical aperture (NA) microscope objective (Nikon Ltd., E Plan 100×, NA 1.25 in oil). A single particle is trapped at a gas pressure of ∼100 mbar, which is reduced to 1 mbar so that the particle motion becomes ballistic. The optical power can be adjusted in the above power range to obtain a desired oscillation frequency from 0.4 to 1.1 kHz.

### High-speed camera imaging

To track the motion of the trapped particle, we use a fast CMOS camera (Mikrotron, EoSens MC1362) with a frame rate of 5000 fps synchronized with nanosecond laser pulses of 532 nm. This allows obtaining stroboscopic images of the trapped particles over a period of 1 s with a bandwidth of 2.5 kHz, which is greater than the trap frequency typically in the range of 0.4 to 1.1 kHz.

### Ultrahigh *Q* experiments

To perform phase- or frequency-locking experiments, the particle motion is tracked by an avalanche photodiode (APD; Thorlabs, APD410C), which measures the forward-scattered light from the trapped particle. The APD signal is processed by a lock-in amplifier (Zurich Instruments, HF2LI, 210 MSa/s, DC-50 MHz) to extract the oscillation frequency and its phase of the particle oscillation. A frequency-doubled waveform with an adjusted phase shift relative to the particle oscillation is superimposed to the voltage waveform driving an acousto-optic modulator (IntraAction, DTD-274HD6M) to modulate the trap intensity (±5%). The PSD is taken over continuous 17.2 hours for phase locking (Fig. 5B) and 30 hours for frequency locking (Fig. 5C).

## Supplementary Material

aaz9858_Movie_S3.mp4

aaz9858_Movie_S2.mp4

aaz9858_Movie_S1.mp4

aaz9858_SM.pdf

## SUPPLEMENTARY MATERIALS

Supplementary material for this article is available at http://advances.sciencemag.org/cgi/content/full/6/23/eaaz9858/DC1
