# Supplementary material for: Coherent oscillations of a levitated birefringent microsphere in vacuum driven by nonconservative rotation-translation coupling
Source: Sci Adv. 2020 Jun 3;6(23):eaaz9858. doi: 10.1126/sciadv.aaz9858 (PMC7269642; doi:10.1126/sciadv.aaz9858)
Supplement: aaz9858_SM.pdf [file aaz9858_SM.pdf]

Science Advances
AAAS

advances.sciencemag.org/cgi/content/full/6/23/eaaz9858/DC1

Supplementary Materials for

Coherent oscillations of a levitated birefringent microsphere in vacuum driven by nonconservative rotation-translation coupling

Yoshihiko Arita*, Stephen H. Simpson*, Pavel Zemánek, Kishan Dholakia*

*Corresponding author. Email: ya10@st-andrews.ac.uk (Y.A.); simpson@isibrno.cz (S.H.S.); kd1@st-andrews.ac.uk (K.D.)

Published 3 June 2020, *Sci. Adv.* **6**, eaaz9858 (2020)
DOI: 10.1126/sciadv.aaz9858

The PDF file includes:

Sections S1 to S5
Figs. S1 to S5
Legends for movies S1 to S3
References

Other Supplementary Material for this manuscript includes the following:

(available at advances.sciencemag.org/cgi/content/full/6/23/eaaz9858/DC1)

Movies S1 to S3

Coherent oscillations of a levitated birefringent microsphere in vacuum driven by non-conservative rotation-translation coupling: Supplementary Information

Yoshihiko Arita,[1,2,*] Stephen H. Simpson,[3,†] Pavel Zemánek,[3] and Kishan Dholakia[1,4,5,6,‡]

[1] *SUPA, School of Physics & Astronomy, University of St Andrews, North Haugh, St Andrews KY16 9SS, United Kingdom*

[2] *Molecular Chirality Research Centre, Chiba University, 1-33 Yayoi-cho, Inage-ku, Chiba-shi 263-0022, Japan*

[3] *Institute of Scientific Instruments of the Czech Academy of Science, v.v.i., Královopolská 147, 612 64 Brno, Czech Republic*

[4] *Graduate School of Engineering, Chiba University, 1-33 Yayoi-cho, Inage-ku, Chiba-shi 263-0022, Japan*

[5] *College of Optical Sciences, The University of Arizona, Tucson, Arizona 85721-0094, USA*

[6] *Department of Physics, College of Science, Yonsei University, Seoul 03722, South Korea*

(Dated: February 12, 2020)

CONTENTS

S1. Vaterite 1

S2. Gas pressure and viscosity in a rarefied gas 2

S3. Numerical simulations 2

S4. Theory for linearised forces with rotation-translation coupling 3
- A. Eigenfrequencies and stability 5
  - 1. Overdamped limit, inertia negligible 6
  - 2. Underdamped, low pressure limit, viscosity negligible 6
  - 3. Critical viscosity giving $\Im(\omega_i) = 0$ 7
- B. Power spectral density 7
- C. Time correlations 9
- D. Specialisation to uncoupled (1D) systems 10

S5. Additional supplementary materials 11

S1. VATERITE

Vaterite is a polymorph of calcium carbonate ($CaCO_3$) with a spherical morphology [Fig. S1(a)] (see Methods section). It is polycrystalline, composed of highly oriented nanocrystals ($20-30$ nm in diameter), in such a way that the optical axes are laid in a hyperbolic distribution throughout the whole vaterite crystal [Fig. S1(b)] [38]. As a result, vaterite is effectively a positive uniaxial birefringent crystal with the birefringence $\Delta n \approx 0.1$, which allows the exchange of spin angular momentum with circularly polarised light [29]. When vaterite is trapped with a linearly polarised beam, on the other hand, the mean optical axis of the crystal will align to the electric field (x axis), which is perpendicular to the propagation direction of the trapping beam (z axis) [Figs. S1(b,c)].

---

* ya10@st-andrews.ac.uk; equal contribution

† simpson@isibrno.cz; equal contribution

‡ kd1@st-andrews.ac.uk

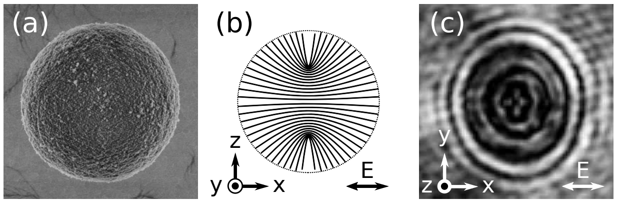

FIG. S1. External and internal structures of vaterite microspheres. (a) SEM image of a synthesised vaterite particle with a radius of 2.2 μm. (b) Schematic showing a hyperbolic distribution of optical axes on a cross-section at the centre of a sphere, where the mean optical axis is in the x-direction. With a linearly polarised beam (z axis), the mean optical axis will align to the direction of $\mathbf{E}$ along the x axis. (c) Optical microscope image of a vaterite particle (2.2 μm in radius) levitated in vacuum with a linearly polarised beam, where the particle orients its optical axis (x-direction) to the $\mathbf{E}$-field. Due to the birefringence, optical image of the particle may look like an oval shape.

S2. GAS PRESSURE AND VISCOSITY IN A RAREFIED GAS

Gas viscosity around a microsphere in a rarefied gas is not the same as the bulk viscosity but depends on the ratio of r/λ, where r is the radius of the particle and λ is the mean free path of air molecules [19]. To directly measure gas viscosity in a rarefied gas, we have trapped a vaterite microsphere of the same size ($r = 2.2$ μm) with a circularly polarised beam. Fig. S2 shows the rotation rate of the microsphere depending on the gas pressure from 1000 mBar (atmospheric) to 1 mBar (blue curve). Calibrating air viscosity of 18.1μPa s at atmospheric pressure, gas viscosity around the microsphere (red curve) can be determined by its rotation rate (blue curve), where we obtain the critical viscosity $\mu_X = 0.2$ μPa s at the critical pressure of $P_X = 1.18$ mBar.

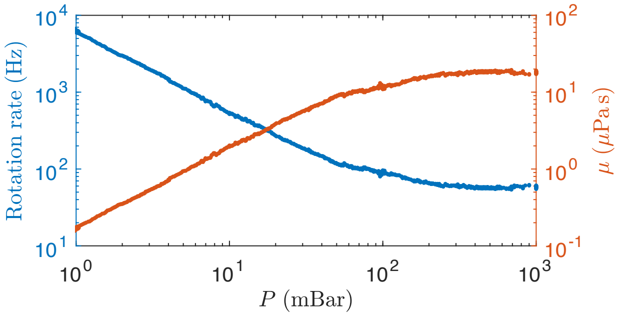

FIG. S2. Gas viscosity determined by the rotation of a vaterite microsphere.

S3. NUMERICAL SIMULATIONS

Direct simulations are performed in 3d by numerical integration of the following Langevin equation,

$$\mathbf{f}^{opt}(\mathbf{q}) + \mathbf{f}^L(t) - mg\hat{\mathbf{z}} - \mathbf{\Xi}\dot{\mathbf{q}} = \mathbf{M}\ddot{\mathbf{q}}, \quad \text{(S1)}$$

where $\mathbf{q}$ are the coordinates of the centre mass (x, y, z) and orientation of the rotationally symmetric particle. $\mathbf{f}^L(t)$ is the uncorrelated Langevin force, with amplitude fixed by the fluctuation-dissipation theorem and a mean value of zero.

$$\langle \mathbf{f}^L(t) \rangle = 0, \quad \text{(S2)}$$

$$\langle \mathbf{f}^L(t) \otimes \mathbf{f}^L(t') \rangle = 2k_B T \mathbf{\Xi} \delta(t - t'). \quad \text{(S3)}$$

$\mathbf{\Xi}$ is the hydrodynamic friction with diagonal components $\xi_{xx} = 6\pi\mu a$, for translations and $\xi_{\theta\theta} = 8\pi\mu a^3$ for rotations. $\mathbf{M}$ the mass (m) and moment of inertia, (I). We use the integration scheme described in [39]. Although this numerical scheme applies only to translational motion, it can be trivially extended to include rotations. Instead of updating positions and velocities alone, we also update orientations and angular velocities. As usual, this is achieved through successive multiplication of rotation matrices (See [40] and references therein). Optical calculations are performed with T-matrix theory [41] and the forces are calculated through integrals of the optical momentum flux, given by the

Quantity	Value
Numerical aperture, NA	0.95
Fill factor, f_0	2.0
Truncation, N_{max}	40
Time step Δt	2×10^{-5} s
No. iterations, N	5×10^4
Sphere radius, a	2.20 µm
Refractive indices (n_e, n_o)	$(1.65, 1.55)$

TABLE S1. Default parameters of the simulation.

Maxwell stress tensor, through a closed surface surrounding the particle. In particular, optical fields are expanded in vector spherical harmonics labelled by eigenvalue pairs (n, m) with $|m| < n$. Expansions are truncated at $n = N_{max}$. The particle itself is modelled as a homogeneous sphere with uniform, birefringent refractive indices [42]. Although this ignores the internal structure of real vaterite particles, it is, nonetheless, a birefringent particle with the same overall symmetry and should be expected to behave in a similar way to a real particle. Numerical simulations are performed with a sphere of nominal parameters corresponding to experimental conditions, i.e. the radius is 2.2 µm, and the ordinary and extraordinary refractive indices are those of bulk vaterite, i.e. ($n_e = 1.65$, $n_o = 1.55$). The modelled beam is a linearly polarised Gaussian beam, rendered by the Richards and Wolf formulation [43], with the same parameters as those used in the experiment. An optical power (i.e. integral of the normal component of the Poynting vector over a plane normal to the beam axis) of 5 mW is used for all simulations. As the simulation runs, a Brownian trail is collected. From this trail we can evaluate power spectral densities and time dependent covariances. Table S1 gives the default parameters of the simulation. Simulations of this sort take $\approx$ 10 hours on a typical desk top computer.

Fig. S3 describes the motion of the centre of mass of the particle. Three conditions are considered, (i) a birefringent particle $\Delta n = 0.1$ at low viscosity $\mu = 1 \times 10^{-6}$ Pa s, (ii) the same birefringent particle at a higher viscosity, $\mu = 3.25 \times 10^{-6}$ Pa s, and (iii) an optically isotropic particle, $\Delta n = 0$ at the lower viscosity, $\mu = 1 \times 10^{-6}$ Pa s. The top left panel shows a scatter plot of the centre of mass in the transverse (xy) plane. The top right shows the power spectral density of the x coordinate of the centre of mass. The lower panel shows the autocorrelation of the x coordinate. As can be seen, cases (ii) and (iii) show similar behaviour; the centre of mass is symmetrically confined by the trap and the power spectral densities show no particularly sharp peaks. The autocorrelation of the x coordinate thermalises quickly. The behaviour for case (i) is dramatically different. As with the experiment, the x coordinate varies much more widely, $\pm \sim 1$ µm, while the y coordinate remains tightly confined. Very sharp peaks appear in the power spectrum, the strongest (by a factor of $\sim 10^6$) close to the resonant frequency of the trap. In addition the oscillation of the x coordinate becomes highly coherent, with very slow decay of the autocorrelation function. These findings closely mimic those observed in the experiment, as discussed above and in the main text.

The simulations allow us to understand the rotational motion of the particle. A strong interaction between the position (x) and pitch (θ) of the birefringent sphere is observed, see Fig. S4. The scatter plot of the (x, θ) coordinates, combined with the cross correlation reveal the mechanism responsible for the formation of the high amplitude coherent oscillations. Vibrations in x couple constructively with vibrations in θ and become self-sustaining. In particular, angular oscillations generate force components in the x direction, that drive the x oscillation. Similarly, the oscillation in x generates torques about the y axis, driving the angular oscillation. Thus, angular and translational oscillations feed one another, passing energy from the optical field to the particle motion. At steady state, this accumulated energy is balanced by viscous dissipation into the gas. The resulting, self-sustaining oscillation is very clearly illustrated in the accompanying supplementary movie 2.

S4. THEORY FOR LINEARISED FORCES WITH ROTATION-TRANSLATION COUPLING

For small displacements, all of the degrees of freedom are uncoupled, except x and θ. This follows from basic symmetry arguments [8,42,44,45], and is confirmed by the T-matrix calculations. Uncoupled degrees of freedom are trivial and, for completeness, treated in the final section below. In summary, uncoupled degrees of freedom are effectively one dimensional and, since any 1D force field can be derived from a potential, they show the usual equilibrium behaviour with the thermal and elastic energies being equal, and the variance of the coordinates independent of viscosity. The anomalous behaviour discussed in this paper comes from the coupling between x and θ. Essential features of the system can be captured by restricting attention to these coupled coordinates.

In the following we consider a linearised Langevin equation for the coupled degrees of freedom of the vaterite

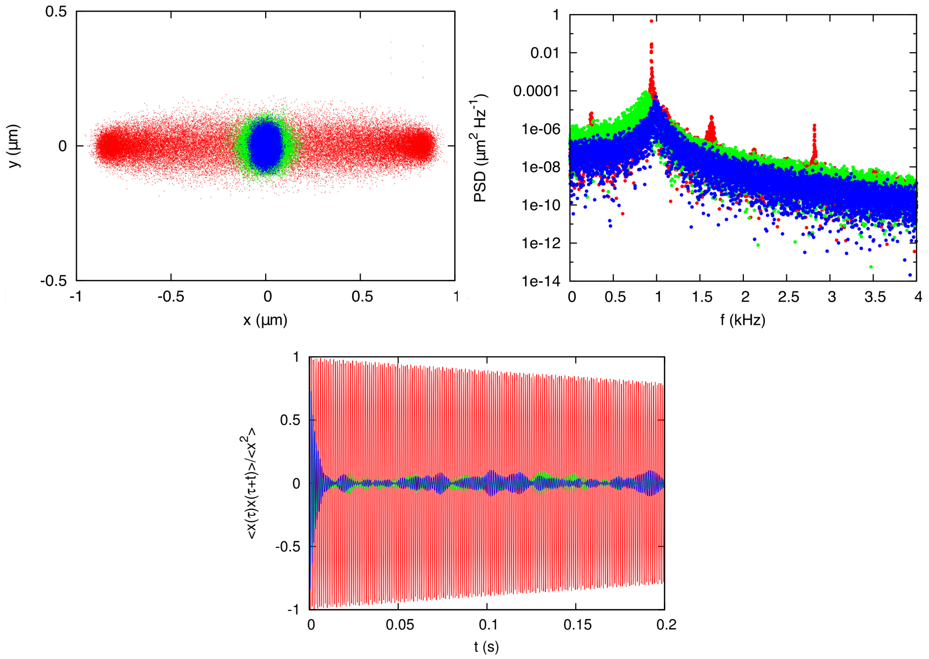

FIG. S3. Simulation data showing the centre of mass motion of the particle under three different conditions, (i) a birefringent particle $\Delta n = 0.1$ at low viscosity $\mu = 1 \times 10^{-6}$ Pa s (shown in red), (ii) the same birefringent particle at a higher viscosity, $\mu = 3.25 \times 10^{-6}$ Pa s (green), (iii) an optically isotropic particle, $\Delta n = 0$ at the lower viscosity, $\mu = 1 \times 10^{-6}$ Pa s (blue). The top left panel shows a scatter plot of the centre of mass in the transverse (xy) plane. The top right shows the power spectral density of the x coordinate of the centre of mass. The lower panel shows the autocorrelation of the x coordinate

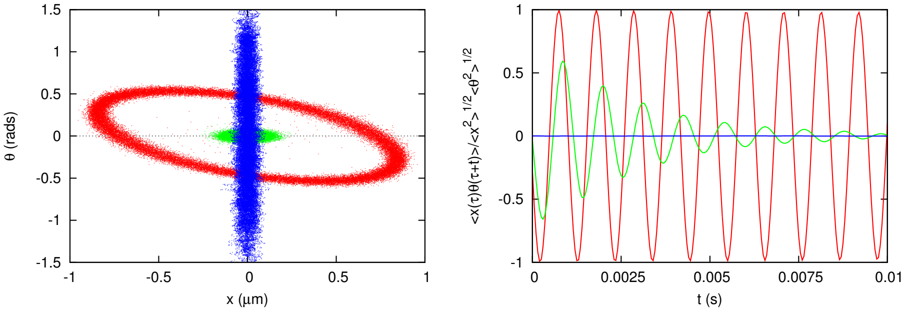

FIG. S4. Simulation data showing the coupling between the x and θ coordinates. The same three conditions, as considered in Fig. S3, are used with the same colours. The left hand panel shows a scatter plot of (x, θ). The right hand panel shows the cross correlations between x and θ.

particle. As discussed below, uncoupled coordinates are effectively at equilibrium and do not play a part in the effects of interest.

$$-\mathbf{K}\mathbf{q} - \boldsymbol{\Xi}\dot{\mathbf{q}} + \mathbf{f}^L = \mathbf{M}\ddot{\mathbf{q}}, \tag{S4}$$

where $\mathbf{K}$ is a stiffness matrix given by linearising the external forces ($\mathbf{f}^{ext} = \mathbf{f}^{opt} - mg\hat{\mathbf{z}}$) about the trapping configuration i.e. $\mathbf{K} = -[\nabla \mathbf{f}^{ext}(\mathbf{q}_{eq})]$, $\boldsymbol{\Xi}$ the hydrodynamic friction, $\mathbf{M}$ the mass (m) and moment of inertia, (I), and $\mathbf{q}$ are the coupled coordinates.

$$\mathbf{K} = \begin{bmatrix} K_{xx} & K_{x\theta} \\ K_{\theta x} & K_{\theta\theta} \end{bmatrix}, \quad \boldsymbol{\Xi} = \begin{bmatrix} \xi_{xx} & 0 \\ 0 & \xi_{\theta\theta} \end{bmatrix}, \quad \mathbf{M} = \begin{bmatrix} m & 0 \\ 0 & I \end{bmatrix}, \quad \mathbf{q} = \begin{pmatrix} x \\ \theta \end{pmatrix}. \tag{S5}$$

Here, the external (i.e. optical and gravitational) forces and torques have been linearly approximated relative to a

position and orientation at which they vanish and are locally restoring. We find the stiffness coefficients by orienting the particle with its optic axis parallel to the x axis, and scanning its centre of mass along the z axis in increments of 0.1 µm. At each stage we calculate the the z component of the force. When it changes sign, we have an interval within which we perform a golden search for the point at which the forces and torques vanish. The stiffness coefficients are then found by numerically calculating the gradient of the forces about the trapping point. For positively birefringent spheres the optic axis aligns with the electric polarisation and the centre of mass lies on the beam axis so that the upward optical scattering forces are equal and opposite to the downward weight of the particle. Optical forces are, in general, non-conservative [6]. As a result $\mathbf{K}$ is generally non-symmetric (i.e. $K_{x\theta} \neq K_{\theta x}$) [8]. $\mathbf{f}^L(t)$ is the uncorrelated Langevin force, with amplitude fixed by the fluctuation-dissipation theorem and a mean value of zero.

$$\langle \mathbf{f}^L(t) \rangle = 0, \tag{S6}$$
$$\langle \mathbf{f}^L(t) \otimes \mathbf{f}^L(t') \rangle = 2k_BT\mathbf{\Xi}\delta(t-t'). \tag{S7}$$

In the frequency domain, this becomes,

$$\Big(\mathbf{K} + i\omega\mathbf{\Xi} - \mathbf{M}\omega^2\Big)\mathbf{Q} = \mathbf{F}^L. \tag{S8}$$

Equivalently,

$$\mathbf{A}\mathbf{Q} = \mathbf{F}^L, \tag{S9a}$$
$$\mathbf{A} = \Big(\mathbf{K} + i\omega\mathbf{\Xi} - \mathbf{M}\omega^2\Big), \tag{S9b}$$
$$= \begin{bmatrix} (K_{xx} + i\omega\xi_{xx} - m\omega^2) & K_{x\theta} \\ K_{\theta x} & (K_{\theta\theta} + i\omega\xi_{\theta\theta} - I\omega^2) \end{bmatrix}, \tag{S9c}$$

where,

$$\langle \mathbf{F}^L(\omega) \rangle = 0, \tag{S10}$$
$$\langle \mathbf{F}^L(\omega) \otimes \mathbf{F}^{L,*}(\omega) \rangle = 2k_BT\mathbf{\Xi}. \tag{S11}$$

The eigenfrequencies of the matrix $\mathbf{A}$ in Eq. (S9) determine the stability of the trap and statistical measures of the stochastic motion of the particle. Below, we describe some general properties of these eigenfrequencies, their dependence on the ambient viscosity and the consequences for the linear stability, power spectral density (PSD) and time correlations of the trapped particle.

A. Eigenfrequencies and stability

The eigenfrequencies of $\mathbf{A}$, $\{\omega_i\}$ are given by the condition $|\mathbf{A}(\omega)| = 0$, a quartic in ω,

$$|\mathbf{A}(\omega)| \equiv P(\omega) = mIP_x(\omega)P_\theta(\omega) - K_{x\theta}K_{\theta x}, \tag{S12}$$

where,

$$P_x(\omega) = \omega^2 - i\frac{\xi_{xx}}{m}\omega - \frac{K_{xx}}{m}, \tag{S13a}$$
$$P_\theta(\omega) = \omega^2 - i\frac{\xi_{\theta\theta}}{I}\omega - \frac{K_{\theta\theta}}{I}. \tag{S13b}$$

Linear stability requires that $\Im(\omega_i) > 0$ for each ω_i. Since $P(\omega)$ is a quartic, succinct expressions for the ω_i are not generally available. However, by examining two extreme conditions, the overdamped limit (where inertia is negligible) and the underdamped limit (where viscosity is negligible), we can anticipate situations in which one of the $\Im(\omega_i)$ will become negative as the ambient viscosity is decreased. Below, we derive this condition and give an expression for the viscosity at which $\Im(\omega_i) = 0$, and associated value of the real part of the frequency.

1. Overdamped limit, inertia negligible

In the low Reynolds number regime, inertia is negligible and Eq. (S12) becomes:

$$P(\omega) = \Bigg((K_{xx} + i\omega\xi_{xx})(K_{\theta\theta} + i\omega\xi_{\theta\theta}) - K_{x\theta}K_{\theta x} \Bigg), \tag{S14}$$

which has roots,

$$\omega = \frac{i}{2}\frac{(K_{xx}\xi_{\theta\theta} + K_{\theta\theta}\xi_{xx})}{\xi_{xx}\xi_{\theta\theta}} \pm i\sqrt{\Delta_{OD}}, \tag{S15}$$

with,

$$\Delta_{OD} = \frac{(K_{xx}\xi_{\theta\theta} - K_{\theta\theta}\xi_{xx})^2}{4\xi_{xx}^2\xi_{\theta\theta}^2} + \frac{K_{x\theta}K_{\theta x}}{\xi_{xx}\xi_{\theta\theta}}. \tag{S16}$$

In order for the trap to be unstable in this regime, we require $\Im(\omega_i) < 0$. From Eq. (S15) this means,

$$\left(\frac{K_{xx}\xi_{xx} + K_{\theta\theta}\xi_{xx}}{2\xi_{xx}\xi_{\theta\theta}} \right)^2 < \left(\frac{K_{xx}\xi_{\theta\theta} - K_{\theta\theta}\xi_{xx}}{2\xi_{xx}\xi_{\theta\theta}} \right)^2 + \frac{K_{x\theta}K_{\theta x}}{\xi_{xx}\xi_{\theta\theta}} \equiv \Delta_{OD}, \tag{S17}$$

which reduces to,

$$K_{x\theta}K_{\theta x} > K_{xx}K_{\theta\theta}. \tag{S18}$$

This is a hard condition to satisfy. The right hand side is positive (if the forces are restoring), which requires comparatively large coupling coefficients of the same sign. In the course of the parametric survey, presented below, this situation never arises. It may be fundamentally impossible for coupling forces and torques to exceed restoring forces and torques in amplitude.

2. Underdamped, low pressure limit, viscosity negligible

In low pressure limit, $\boldsymbol{\Xi} \to 0$ and Eq. (S12) becomes,

$$P(\omega) = mI\Bigg(\Big(\omega^2 - \frac{K_{xx}}{m}\Big)\Big(\omega^2 - \frac{K_{\theta\theta}}{I}\Big) - \frac{K_{x\theta}K_{\theta x}}{mI} \Bigg). \tag{S19}$$

In this case, the roots of $P(\omega) = 0$ are,

$$\omega^2 = \frac{1}{2}\left(\frac{K_{\theta\theta}}{I} + \frac{K_{xx}}{m} \right) \pm \sqrt{\Delta_{LP}} \tag{S20}$$

with,

$$\Delta_{LP} = \frac{1}{4}\left(\frac{K_{\theta\theta}}{I} - \frac{K_{xx}}{m} \right)^2 + \frac{K_{x\theta}K_{\theta x}}{mI}. \tag{S21}$$

Owing to the inclusion of inertia, the number of eigenfrequencies is twice that in the overdamped limit.
Without x/θ coupling, we get:

$$\omega_1^2 = \tfrac{K_{xx}}{m}, \qquad \omega_2^2 = \tfrac{K_{\theta\theta}}{I}, \tag{S22}$$

i.e. the spring vibrates at its natural frequencies. In the underdamped limit, $\Im(\omega_i) < 0$ for at least one of the $\{\omega_i\}$ whenever $\Delta_{LP} < 0$. Compared to the overdamped regime, this condition is relatively easily satisfied. For example, if

the resonant frequencies for the x and θ oscillations are close, the bracketed term approaches zero and the stability condition reduces to $K_{x\theta}K_{\theta x} < 0$. In this case, the size of the coupling coefficients is immaterial, it is only necessary that they are of opposite sign.

3. *Critical viscosity giving* $\Im(\omega_i) = 0$

When the trap is stable with respect to rotation-translation coupling in the overdamped limit, but unstable in the underdamped limit then the value of at least one of the $\Im(\omega_i)$ must change from positive to negative as the viscosity is decreased. The critical viscosity, for which $\Im(\omega_i) = 0$, can be determined, by requiring that $\omega_i = \Re(\omega_i) = \omega_r$ is purely real in Eq. (S12). Under this condition, the real and imaginary parts of $P(\omega)$ must both be zero,

$$mI\omega_r^4 - (mK_{\theta\theta} + IK_{xx} + \xi_{xx}\xi_{\theta\theta})\omega_r^2 + (K_{xx}K_{\theta\theta} - K_{x\theta}K_{\theta x}) = 0, \tag{S23a}$$

$$-(m\xi_{\theta\theta} + I\xi_{xx})\omega_r^3 + (K_{xx}\xi_{\theta\theta} + K_{\theta\theta}\xi_{xx})\omega_r = 0. \tag{S23b}$$

Note that, according to Eqs. (S12,S13), when $K_{x\theta} = K_{\theta x} = 0$, the condition $\Im(\omega) = 0$ requires $\frac{\xi_{xx}}{m}\omega_r = 0$ or $\frac{\xi_{\theta\theta}}{I}\omega_r = 0$ which when $\xi_{xx} = 0$ or $\xi_{\theta\theta} = 0$, which can only be satisfied when the viscosity and, therefore, the pressure are zero. When $K_{x\theta} \neq 0$ and $K_{\theta x} \neq 0$, Eqs. (S23a,b) must hold simultaneously: Eq. (S23b) gives,

$$\omega_X^2 = \frac{K_{xx}\xi_{\theta\theta} + K_{\theta\theta}\xi_{xx}}{m\xi_{\theta\theta} + I\xi_{xx}}, \tag{S24}$$

where ω_X is the real frequency at which the imaginary component vanishes. Since both ξ_{xx} and $\xi_{\theta\theta}$ are directly proportional to the viscosity, i.e. $\xi_{xx} = 6\pi\mu a \equiv s_x\mu$, $\xi_{\theta\theta} = 8\pi\mu a^3 \equiv s_\theta\mu$,

$$\omega_X^2 = \frac{K_{xx}s_\theta + K_{\theta\theta}s_x}{ms_\theta + Is_x}. \tag{S25}$$

This expression can be substituted into Eq. (S23a) to give a relationship for the corresponding critical viscosity, μ_X.

$$mI\omega_X^4 - (mK_{\theta\theta} + IK_{xx} + \xi_{xx}\xi_{\theta\theta})\omega_X^2 + (K_{xx}K_{\theta\theta} - K_{x\theta}K_{\theta x}) = 0, \tag{S26}$$

rearranging,

$$\mu_X^2 s_x s_\theta = mI\omega_X^2 - (mK_{\theta\theta} + IK_{xx}) + (K_{xx}K_{\theta\theta} - K_{x\theta}K_{\theta x})/\omega_X^2, \tag{S27}$$

where μ_X is the critical viscosity for which one of the $\Im(\omega_i) = 0$. When $K_{x\theta} = K_{\theta x} = 0$ this expression gives a negative μ_X^2. The product $K_{x\theta}K_{\theta x}$ must be negative enough to make μ^2 positive. Interestingly, $\omega_X = \Re(\omega_i)$ is completely independent of viscosity and given as a function of the stiffness coefficients, $K_{xx}, K_{\theta\theta}$, the radius of the particle, its mass and moment of inertia. For a fixed field and particle, optical forces are directly proportional to the optical power. It may seem appear that ω_X^2 and μ_X are directly proportional to the beam power however this is only approximately correct. The trapping configuration of the particle is determined by a balance between the downward weight and the upward scattering force. As the power in the beam changes, so does the height of the trapping configuration. For this reason the stiffness in the trapping configuration is not directly proportional to the beam power.

Since ω_X and μ_X can be simply calculated from the stiffness parameters at the equilibrium trapping configuration, we can efficiently assess their variation with respect to the parameters defining the trap. Fig. S5 shows calculated values of ω_X and μ_X as functions of anisotropy, $\Delta n = n_e - n_o$ for varying beam power, sphere radius, mean refractive index and beam numerical aperture. The mean refractive index, $\langle n \rangle^2 = (n_e^2 + 2n_o^2)/3$, is held constant, unless its variation is being considered.

In general, μ_X increases rapidly with Δn, providing that $\Delta_{LP} < 0$, and varies in the range $0 \to\approx 5 \times 10^{-6}$ Pa s. ω_X can vary from around 400 Hz to 1500 Hz within the range of parameters considered here. The strongest dependence of ω_X is with the beam power.

B. Power spectral density

The power spectral density (PSD) for the coupled system can be expressed in terms of the eigenfrequencies, ω_i When the preceeding conditions are satisfied, and one of the $\Im(\omega_i) \to 0$ as $\mu \to \mu_X$, then the corresponding motional mode (or eigenvector of the motion) begins to dominate. Measures of the stochastic motion of the particle can then be

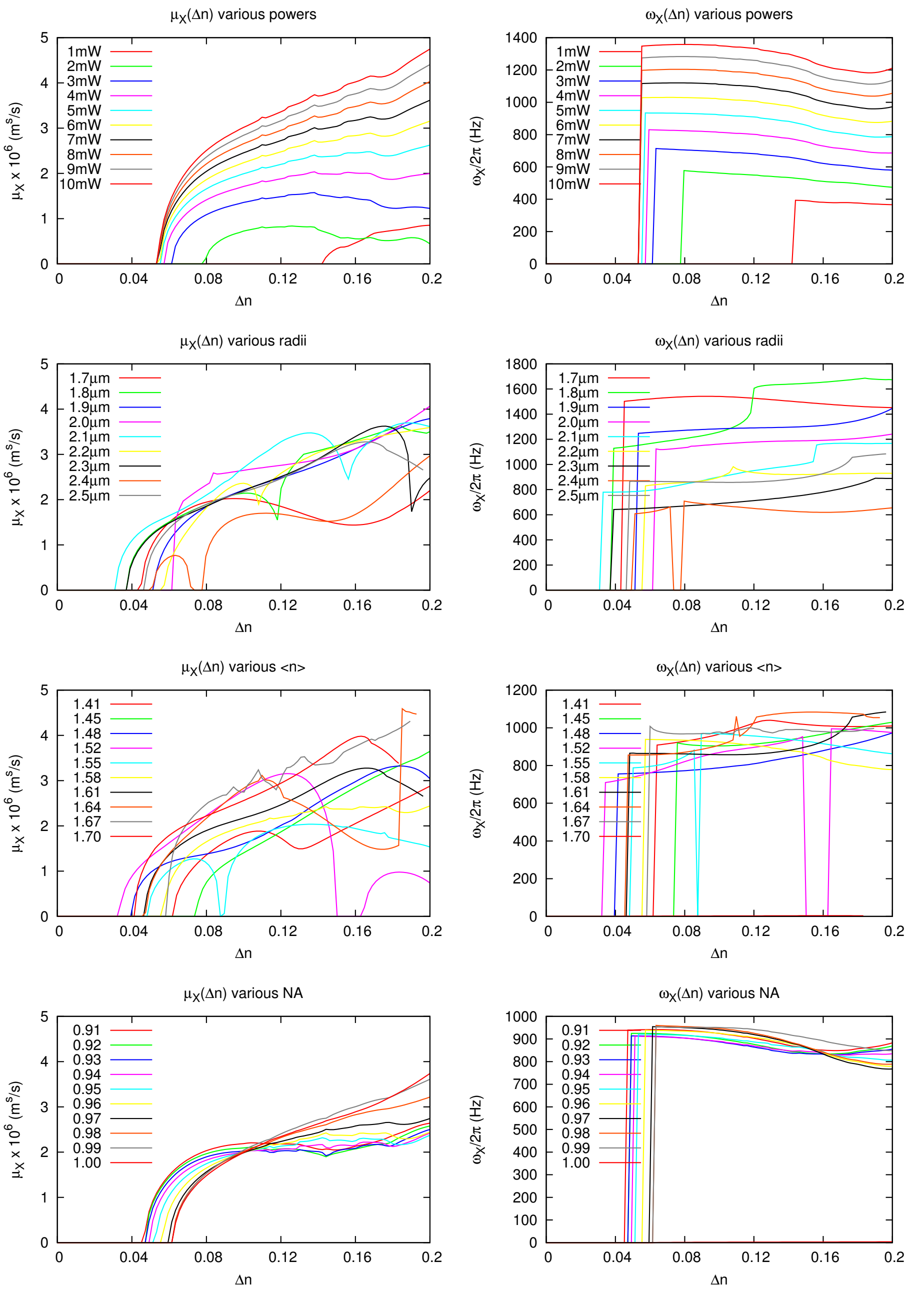

FIG. S5. Parametric survey of the critical viscosities, μ_X, and frequencies, ω_X. Plots of μ_X (left column) and ω_X (right column) versus anisotropy are shown. Individual curves in *Row 1* correspond to different beam powers in the range 1 mW to 10 mW. *Row 2* curves correspond to different particle radii in the range 1.7 µm to 2.5 µm. *Row 3* curves correspond to average refractive indices in the range 1.41 to 1.7. *Row 4* curves correspond to numerical apertures in the range 0.9 to 1.

expressed in terms of this mode alone. Use this definition from now on **Power spectra:** from above,

$$\langle \mathbf{Q}(\omega) \otimes \mathbf{Q}^*(\omega) \rangle = \frac{2k_B T}{P(\omega)P^*(\omega)} \mathbf{C}(\omega)\mathbf{\Xi}\mathbf{C}^H(\omega), \tag{S28}$$

where,

$$\mathbf{A}^{-1} = \frac{-1}{P(\omega)} \begin{bmatrix} IP_\theta(\omega) & K_{x\theta} \\ K_{\theta x} & mP_x(\omega) \end{bmatrix} \equiv -\frac{\mathbf{C}}{P(\omega)}, \tag{S29}$$

and $\mathbf{A}$ is defined in Eq. (S9). The product, $\mathbf{C\Xi C}^H$, is,

$$\mathbf{C}(\omega)\mathbf{\Xi}\mathbf{C}^H(\omega) = \begin{bmatrix} \left(I^2\xi_{xx}P_\theta P_\theta^* + \xi_{\theta\theta}K_{x\theta}^2\right) & \left(I\xi_{xx}K_{\theta x}P_\theta + m\xi_{\theta\theta}K_{x\theta}P_x^*\right) \\ \left(I\xi_{xx}K_{\theta x}P_\theta^* + m\xi_{\theta\theta}K_{x\theta}P_x\right) & \left(m^2\xi_{\theta\theta}P_xP_x^* + \xi_{xx}K_{\theta x}^2\right) \end{bmatrix}, \tag{S30}$$

where P_x, P_θ are the polynomials given in Eq. (S13). The denominator of Eq. (S28), $P(\omega)P^*(\omega)$, determines the general form of the PSD and can be factorized as follows. First, the quartic, $P(\omega)$, can be expressed as, $P(\omega) = \sum i^n a_n \omega^n$, with real coefficients a_n. It follows immediately that, if ω_i is a root of $P(\omega)$, then $-\omega_i^*$ is also a root. For example, if $P(\omega_i) = 0$ then, $P(-\omega_i^*) = \sum i^n a_n(-\omega^*)^n = \sum(-i)^n a_n(\omega_i^*)^n) = \left(\sum i^n a_n \omega_i^n\right)^* = 0$. Similarly, if ω_i is a root of $P(\omega)$, then $-\omega_i$ is a root of $P^*(\omega)$. The roots of $P(\omega)P^*(\omega)$ therefore have the following form: $\{\omega_i, -\omega_i^*, -\omega_i, \omega_i^*\}$ for $i = 1, 2$. Hence, we can write $P(\omega)P^*(\omega)$ as follows,

$$P(\omega)P^*(\omega) = m^2I^2 \prod_{i=1,2} \left(\omega^2 - \omega_i^2\right)\left(\omega^2 - (\omega_i^*)^2\right). \tag{S31}$$

It follows directly that $P(\omega)P^*(\omega) = P(-\omega^*)P^*(-\omega^*)$. We note that similar arguments can be made to show that $\left[\mathbf{C}(\omega)\mathbf{\Xi}\mathbf{C}^H(\omega)\right]^* = \mathbf{C}(-\omega^*)\mathbf{\Xi}\mathbf{C}^H(-\omega^*)$, as can be confirmed directly from Eqns. (S13,S30).

The form of $P(\omega)P^*(\omega)$ fixes the behaviour of the power spectrum for the coupled system. If $\Im(\omega_i) \to 0$ as the viscosity, $\mu \to \mu_X$, then the denominator, $P(\omega)P^*(\omega)$ becomes singular as $\omega \to \omega_X$. In particular, as $\mu \to \mu_X$, $\omega_i \approx \omega_X + i\delta$, for small δ, and $(\omega - \omega_i^2) \approx 2\omega_X\delta i$. As $\mu \to \mu_X$, and $\omega \to \omega_X$,

$$\langle \mathbf{Q}(\omega) \otimes \mathbf{Q}^*(\omega)\rangle \propto \frac{2k_BT}{4\omega_X^2\delta^2}\mathbf{C}(\omega)\mathbf{\Xi}\mathbf{C}^H(\omega). \tag{S32}$$

Qualitatively, as the critical viscosity is approached, the power spectrum is increasingly dominated by a sharp peak at $\omega = \omega_X$, giving an oscillation with a high quality factor at a finite viscosity. As discussed below (section S4 D, Eq. (S41), the behaviour for uncoupled or conservative systems, is similar except that the condition required for the imaginary eigenfrequencies to approach zero is $\mu \to 0$. For coupled systems, a similar limit is reached for finite viscosities, μ_X. More fundamental differences between coupled (non-conservative systems) and uncoupled (effectively conservative systems) are revealed in the correlations and variances of the particles, discussed immediately below.

C. Time correlations

The time correlations follow from the Weiner-Khinchine theorem:

$$\langle \mathbf{q}(t) \otimes \mathbf{q}(t+\tau)\rangle = \frac{1}{2\pi}\int_{-\infty}^{\infty} \langle \mathbf{Q}(\omega) \otimes \mathbf{Q}^*(\omega)\rangle e^{-i\omega\tau} d\omega, \tag{S33}$$

where $\langle \mathbf{Q}(\omega) \otimes \mathbf{Q}^*(\omega)\rangle$ given by Eq. (S28). The integral in Eq. (S33) can be evaluated by applying the residue theorem to a contour closed by a semi-circle in the lower half plane for $\tau > 0$ i.e.

$$\langle \mathbf{q}(t) \otimes \mathbf{q}(t+\tau)\rangle = \frac{1}{2\pi}\oint \langle \mathbf{Q}(\omega) \otimes \mathbf{Q}^*(\omega)\rangle e^{-i\omega\tau} d\omega = i\sum_j Res\left(\langle \mathbf{Q}(\omega) \otimes \mathbf{Q}^*(\omega)\rangle e^{-i\omega\tau}, \omega_j\right), \tag{S34}$$

where ω_j are the poles in the lower half plane. If ω_1 is one such pole, the corresponding residue is,

$$\begin{aligned} Res\left(\langle \mathbf{Q}(\omega) \otimes \mathbf{Q}^*(\omega)\rangle e^{-i\omega\tau}, \omega_1\right) &= \lim_{\omega\to\omega_1} 2k_BT\frac{(\omega-\omega_1)}{P(\omega)P^*(\omega)}\mathbf{C}(\omega)\mathbf{\Xi}\mathbf{C}^H(\omega)e^{-i\omega\tau} \\ &= \frac{k_BT\mathbf{C}(\omega_1)\mathbf{\Xi}\mathbf{C}^*(\omega_1)e^{-i\omega_1\tau}}{4m^2I^2\Re(\omega_1)\Im(\omega_1)\omega_1(\omega_1^2-\omega_2^2)(\omega_1^2-(\omega_2^*)^2)}. \end{aligned} \tag{S35}$$

As discussed in section (S4 B), if ω_1 is a pole in the lower half plane, then so is $-\omega_1^*$, and $\mathbf{C}(\omega)\mathbf{\Xi}\mathbf{C}^H(\omega) = \mathbf{C}(-\omega^*)\mathbf{\Xi}\mathbf{C}^H(\omega^*)$. It follows that,

$$Res\left(\langle \mathbf{Q}(\omega) \otimes \mathbf{Q}^*(\omega)\rangle e^{-i\omega\tau}, -\omega_1^*\right) = -\left[Res\left(\langle \mathbf{Q}(\omega) \otimes \mathbf{Q}^*(\omega)\rangle e^{-i\omega\tau}, \omega_1\right)\right]^*. \tag{S36}$$

Summing the residues at $\omega = \omega_1$ and $\omega = -\omega_1^*$ gives,

$$Res\big(\langle \mathbf{Q}(\omega) \otimes \mathbf{Q}^*(\omega)\rangle e^{-i\omega\tau}, \omega_1\big) + Res\big(\langle \mathbf{Q}(\omega) \otimes \mathbf{Q}^*(\omega)\rangle e^{-i\omega\tau}, -\omega_1^*\big) = \\ 2i\Im\Big[Res\big(\langle \mathbf{Q}(\omega) \otimes \mathbf{Q}^*(\omega)\rangle e^{-i\omega\tau}, \omega_1\big)\Big]. \quad \text{(S37)}$$

There must be a further two more poles in the lower half plane, either $\omega_2, -\omega_2^*$, or $-\omega_2, \omega_2^*$, the residues of which can be summed in a similar way.

Equations (S35,S37), show the key point. The covariance matrix, $\langle \mathbf{q}(t) \otimes \mathbf{q}(t+\tau)\rangle$ is the sum of two damped oscillations with amplitudes proportional to the reciprocal of the imaginary parts of the eigenfrequencies of the system. As described above, in section S4 A, $\Im(\omega_i) \to 0$, under particular conditions. When these conditions pertain, the time dependent covariance of the coordinates becomes increasingly dominated by this mode as $\mu \to \mu_X$. Both the amplitude (the instantaneous covariance) of this oscillation and its decay time become large. In particular, if $\omega \approx \omega_X + i\delta$ as $\mu \to \mu_X$, we have,

$$\langle \mathbf{q}(t) \otimes \mathbf{q}(t+\tau)\rangle \underset{\sim}{\propto} \frac{k_B T}{\omega_X \delta} e^{-i\omega_X \tau} e^{-\delta\tau}. \quad \text{(S38)}$$

To clarify the meaning of Eq. (S38), we note that $\langle \mathbf{q}(t) \otimes \mathbf{q}(t+\tau)\rangle$ is, of course, a symmetric matrix. This matrix consists of the sum of the residues detailed above. Each term in this sum is weighted by a scalar whose denominator contains a term $\Im(\omega_i)$, and whose time variation if $e^{-i\Re(\omega_i)\tau}e^{-\Im(\omega_i)\tau}$, for characteristic frequencies ω_i with poles in the lower half plane. As $\mu \to \mu_X$, this sum is increasingly dominated by the terms for which $\Im(\omega_i)$ tends to zero. The right hand side of Eq. (S38) shows the limiting form of the scalar function multiplying the well behaved matrix term. The equation shows how each component of the time dependent covariance matrix grows as $\mu \to \mu_X$. This result is fundamentally different from the familiar situation where coordinates are uncoupled (see final section, S4 D) Eq. (S48)). In that case, the amplitude of the time dependent covariance (e.g. $\langle x^2\rangle$) is a constant, independent of viscosity, given by the equipartition theorem e.g. $\frac{1}{2}k_B T = \frac{1}{2}K_{xx}\langle x^2\rangle$ [12]. The decay times of the covariance, meanwhile, also increase with decreasing viscosity but they do so much more slowly, and they do not approach zero for any finite viscosity.

We note that the result, $\langle x^2\rangle \to \infty$, with $\mu \to \mu_X$, obtained for the coupled system, does not indicate that the particle amplitude is becomes unbounded in reality. Physically, the particle makes excursions of increasingly large amplitude until the linear range of the trap is exceeded. Beyond this limit, the motion becomes erratic, or even chaotic. In the experiment, the particle may simply be lost.

In the next section we show that the treatment for the coupled system reduces to the familiar results for uncoupled coordinates when $K_{x\theta} = K_{\theta x} = 0$.

D. Specialisation to uncoupled (1D) systems

Finally, we consider the more familiar case of uncoupled (i.e. one dimensional) systems. In contrast to the coupled systems described above, a one dimensional force field is necessarily conservative. The most conspicuous consequence of this, is that the instantaneous variance of the coordinates, $\langle x^2\rangle$ is independent of viscosity and given by the equipartition theorem, i.e. $\frac{1}{2}k_B T = \frac{1}{2}K_{xx}\langle x^2\rangle$. Other qualitative differences appear in the PSD and the time dependent covariance. In both the coupled and uncoupled cases, the peak in the PSD is $\propto 1/\Im(\omega_i)$ and the decay of the time dependent covariance is $\propto exp(\Im(\omega_i)\tau)$. However, for the uncoupled case, $\Im(\omega_i) \propto \xi_{xx}$, which remains finite for finite viscosity, in contrast to the coupled case for which $\Im(\omega_i) \to 0$ as the viscosity approaches its critical value, μ_X. In the uncoupled case, therefore, the peak in the PSD has a limited height and the time dependent covariance decays more rapidly [Eq. (S48), below], in comparison to the coupled system [Eq. (S38)]. In the following, we show that these familiar results follow from the preceding treatment of the coupled system, when the coupling coefficients are set to zero, i.e. when $K_{x\theta} = K_{\theta x} = 0$.

First of all, the eigenfrequencies are given by the roots of $P(\omega)$, Eq. (S12). When $K_{x\theta} = K_{\theta x} = 0$,

$$P(\omega) = P_x(\omega)P_\theta(\omega), \quad \text{(S39)}$$

so the roots are given by $P_x(\omega) = 0$ and $P_\theta(\omega) = 0$. Restricting attention to the x coordinate, θ being similar, the roots of $P_x(\omega) = 0$ are,

$$\omega_1 = \frac{1}{2m}\Big(i\xi_{xx} \pm \sqrt{4mK_{xx} - \xi_{xx}^2}\Big). \quad \text{(S40)}$$

Notably, $\Im(\omega_1) = \xi_{xx}/2m$, which remains finite for finite viscosity, in contrast to the coupled case.

The PSD, given by Eq. (S28), for the x coordinate is,

$$\langle X(\omega)X^*(\omega)\rangle = \frac{2k_BT}{m^2I^2}\frac{I^2\xi_{xx}P_\theta P_\theta^* + \xi_{\theta\theta}K_{x\theta}^2}{(P_xP_\theta - K_{x\theta}K_{\theta x})(P_x^*P_\theta^* - K_{x\theta}K_{\theta x})} \tag{S41}$$

$$= \frac{2k_BT}{m^2}\frac{\xi_{xx}}{P_xP_x^*}, \tag{S42}$$

when $K_{x\theta} = K_{\theta x} = 0$. Factorizing $P_xP_x^*$ gives the PSD,

$$\langle X(\omega)X^*(\omega)\rangle = \frac{2k_BT}{m^2}\frac{\xi_{xx}}{(\omega^2 - \omega_1^2)(\omega^2 - (\omega_1^*)^2)}. \tag{S43}$$

As the viscosity is reduced, ω_1 becomes,

$$\omega_1 \approx \pm\sqrt{\frac{K_{xx}}{m}} + i\frac{\xi_{xx}}{2m} \equiv \pm\omega_0 + i\frac{\xi_{xx}}{2m}, \tag{S44}$$

where ω_0 is the natural, resonant frequency. Then, the PSD peak height is,

$$\langle X(\omega)X^*(\omega)\rangle_{max} \approx \frac{4k_BT}{\xi_{xx}}, \tag{S45}$$

which becomes large as $\mu \to 0$, but much less rapidly than the coupled case, which becomes large as the viscosity approaches a finite, critical value namely μ_X. In particular the peak height of the PSD $\propto 1/\mu$ for the uncoupled case, and $\propto 1/(\mu - \mu_X)^2$ for the vaterite spheres discussed in this article [see Eq. (S32)].

The time dependent covariance, $\langle x(t)x(t+\tau)\rangle$, depends on the residues of the PSD, through Eq. (S34). Here, the poles in the lower half plane are the roots of P_x^*, i.e. $-\omega_1, \omega_1^*$. The residue at $-\omega_1$ is,

$$Res\big(\langle X(\omega)X^*(\omega)\rangle e^{-i\omega\tau}, -\omega_1\big) = -\frac{2k_BT}{m^2}\frac{\xi_{xx}}{8i\omega_1\Re(\omega_1)\Im(\omega_1)}, \tag{S46}$$

$$= \frac{k_BT}{m}\frac{i\omega_1\xi_{xx}}{|\omega_1|^2\Re(\omega_1)\Im(\omega_1)}. \tag{S47}$$

Summing the residues with Eqs. (S36, S37), and combining with the chracteristic frequency, Eq. (S40) the time correlations, [Eq. (S34)] gives,

$$\langle x(t)x(t+\tau)\rangle = -\frac{k_BT}{m^2}\frac{\xi_{xx}}{2|\omega_1|^2\Im(\omega_1)}e^{i\omega_1\tau} = \frac{k_BT}{K_{xx}}e^{i\omega_1\tau} \approx \frac{k_BT}{K_{xx}}e^{\pm\omega_0\tau}e^{-\frac{\xi_{xx}}{2m}\tau}. \tag{S48}$$

The final expression uses the approximation to the characteristic frequency, Eq. (S44). In terms of algebra, the reason that the instantaneous variance, $\langle x^2\rangle$, is independent of viscosity is that the residues introduce a factor $\Im(\omega_i)$ in the denominator which, in this case is ξ_{xx} [Eq. (S40)]. This factor precisely cancels the term ξ_{xx} that appears in the numerator due to the normalization of the stochastic forces [Eq. (S3)] which itself is a consequence of the fluctuation-dissipation theorem. This cancellation does not generally occur when degrees of freedom are coupled; in that case we have $\Im(\omega_i) \to 0$ at finite viscosities, which cannot cancel the ξ_{xx} in the numerator which, of course, is finite for finite viscosity. Physically, the qualitative difference between the two forms of behaviour rests in the fact that the coupled system is generally non-conservative, and the uncoupled system is effectively one dimensional and, therefore, effectively conservative.

S5. ADDITIONAL SUPPLEMENTARY MATERIALS

Supplementary movie 1 shows a vaterite microsphere (2.2 µm in radius) oscillating along the polarisation direction with an amplitude ≤ 1 µm at a gas pressure of 1.2 mBar. The frame rate is rendered at 15 fps from 5000 fps.

Supplementary movie 2 shows the simulated stochastic motion of optically trapped spheres. The spheres are shown full size. The double arrow bisecting each sphere shows the direction of the symmetry axis each particle. In the case of the isotropic particle, this axis is arbitrarily fixed in the sphere. The parameters used correspond to those

used in Fig. 4, i.e. (red) Birefringent: $\Delta n = 0.1$, lower viscosity: $\mu = 1 \times 10^{-6}$ Pa s, (green) Birefringent: $\Delta n = 0.1$, higher viscosity: $\mu = 3.25 \times 10^{-6}$ Pa s, (blue) Isotropic: $\Delta n = 0$, lower viscosity: $\mu = 1 \times 10^{-6}$ Pa s.

Supplementary movie 3 shows the same stochastic trajectories as supplementary movie 2. To make the rotation-translation coupling clearer we show only the position and orientation of the centre of each particle. This is indicated by a small, 1 μm diameter, circle with a double arrow parallel to the symmetry axis.

REFERENCES AND NOTES

1. I. A. Martínez, E. Roldán, L. Dinis, R. A. Rica, Colloidal heat engines: A review. *Soft Matter* **13**, 22–36 (2017).

2. P. Hänggi, F. Marchesoni, F. Nori, Brownian motors. *Ann. Phys.* **14**, 51–70 (2005).

3. J. Howard, Molecular motors: Structural adaptations to cellular functions. *Nature* **389**, 561–567 (1997).

4. P. Zemánek, G. Volpe, A. Jonáš, O. Brzobohatý, Perspective on light-induced transport of particles: From optical forces to phoretic motion. *Adv. Opt. Photonics* **11**, 577–678 (2019).

5. L. P. Faucheux, L. S. Bourdieu, P. D. Kaplan, A. J. Libchaber, Optical thermal ratchet. *Phys. Rev. Lett.* **74**, 1504–1507 (1995).

6. S. Sukhov, A. Dogariu, Non-conservative optical forces. *Rep. Prog. Phys.* **80**, 112001 (2017).

7. Y. Roichman, B. Sun, A. Stolarski, D. G. Grier, Influence of nonconservative optical forces on the dynamics of optically trapped colloidal spheres: The fountain of probability. *Phys. Rev. Lett.* **101**, 128301 (2008).

8. S. H. Simpson, S. Hanna, First-order nonconservative motion of optically trapped nonspherical particles. *Phys. Rev. E* **82**, 031141 (2010).

9. A. Irrera, A. Magazzú, P. Artoni, S. H. Simpson, S. Hanna, P. H. Jones, F. Priolo, P. G. Gucciardi, O. M. Maragó, Photonic torque microscopy of the nonconservative force field for optically trapped silicon nanowires. *Nano Lett.* **16**, 4181–4188 (2016).

10. W. J. Toe, I. Ortega-Piwonka, C. N. Angstmann, Q. Gao, H. H. Tan, C. Jagadish, B. I. Henry, P. J. Reece, Nonconservative dynamics of optically trapped high-aspect-ratio nanowires. *Phys. Rev. E* **93**, 022137 (2016).

11. G. M. Gibson, J. Leach, S. Keen, A. J. Wright, M. J. Padgett, Measuring the accuracy of particle position and force in optical tweezers using high-speed video microscopy. *Opt. Express* **16**, 14561–14570 (2008).

12. T. C. Li, S. Kheifets, D. Medellin, M. G. Raizen, Measurement of the instantaneous velocity of a Brownian particle. *Science* **328**, 1673–1675 (2010).

13. S. H. Simpson, S. Hanna, Thermal motion of a holographically trapped SPM-like probe. *Nanotechnology* **20**, 395710 (2009).

14. J. Ahn, Z. Xu, J. Bang, P. Ju, X. Gao, T. Li, Ultrasensitive torque detection with an optically levitated nanorotor. *Nat. Nanotechnol.* **15**, 89–93 (2020).

15. S. Kuhn, B. A. Stickler, A. Kosloff, F. Patolsky, K. Hornberger, M. Arndt, J. Millen, Optically driven ultra-stable nanomechanical rotor. *Nat. Commun.* **8**, 1670 (2017).

16. A. Pontin, N. Bullier, M. Toroš, P. Barker, An ultra-narrow line width levitated nano-oscillator for testing dissipative wavefunction collapse. arXiv:1907.06046 (2019).

17. D. E. Chang, C. Regal, S. B. Papp, D. Wilson, J. Ye, O. Painter, H. J. Kimble, P. Zoller, Cavity opto-mechanics using an optically levitated nanosphere. *Proc. Natl. Acad. Sci. U.S.A.* **107**, 1005–1010 (2010).

18. J. Gieseler, L. Novotny, R. Quidant, Thermal nonlinearities in a nanomechanical oscillator. *Nat. Phys.* **9**, 806–810 (2013).

19. S. Beresnev, V. Chernyak, G. Fomyagin, Motion of a spherical particle in a rarefied gas. Part 2. Drag and thermal polarization. *J. Fluid Mech.* **219**, 405–421 (1990).

20. D. Chang, K. Ni, O. Painter, H. Kimble, Ultrahigh-*Q* mechanical oscillators through optical trapping. *New J. Phys.* **14**, 045002 (2012).

21. A. Pikovsky, M. Rosenblum, J. Kurths, *Synchronization: A Universal Concept In Nonlinear Sciences* (Cambridge Univ. Press, 2001).

22. J. Gieseler, M. Spasenović, L. Novotny, R. Quidant, Nonlinear mode coupling and synchronization of a vacuum-trapped nanoparticle. *Phys. Rev. Lett.* **112**, 103603 (2014).

23. V. Svak, O. Brzobohatỳ, M. Šiler, P. Jákl, J. Kaňka, P. Zemánek, S. Simpson, Transverse spin forces and non-equilibrium particle dynamics in a circularly polarized vacuum optical trap. *Nat. Commun.* **9**, 5453 (2018).

24. S. H. Simpson, S. Hanna, T. J. Peterson, G. A. Swartzlander, Optical lift from dielectric semicylinders. *Opt. Lett.* **37**, 4038–4040 (2012).

25. O. Ilic, I. Kaminer, B. Zhen, O. D. Miller, H. Buljan, M. Soljačić, Topologically enabled optical nanomotors. *Sci. Adv.* **3**, e1602738 (2017).

26. D. B. Phillips, M. J. Padgett, S. Hanna, Y. L. D. Ho, D. M. Carberry, M. J. Miles, S. H. Simpson, Shape-induced force fields in optical trapping. *Nat. Photon.* **8**, 400–405 (2014).

27. X. Li, J. Chen, Z. Lin, J. Ng, Optical pulling at macroscopic distances. *Sci. Adv.* **5**, eaau7814 (2019).

28. O. N. Kirillov, *Nonconservative Stability Problems of Modern Physics* (De Gruyter, 2013).

29. Y. Arita, M. Mazilu, K. Dholakia, Laser-induced rotation and cooling of a trapped microgyroscope in vacuum. *Nat. Commun.* **4**, 2374 (2013).

30. T. M. Hoang, Y. Ma, J. Ahn, J. Bang, F. Robicheaux, Z. Q. Yin, T. C. Li, Torsional optomechanics of a levitated nonspherical nanoparticle. *Phys. Rev. Lett.* **117**, 123604 (2016).

31. M. Mazilu, Y. Arita, T. Vettenburg, J. M. Auñón, E. M. Wright, K. Dholakia, Orbital-angular-momentum transfer to optically levitated microparticles in vacuum. *Phys. Rev. A* **94**, 053821 (2016).

32. Y. Arita, M. Chen, E. M. Wright, K. Dholakia, Dynamics of a levitated microparticle in vacuum trapped by a perfect vortex beam: Three-dimensional motion around a complex optical potential. *J. Opt. Soc. Am. B* **34**, C14–C19 (2017).

33. Y. Arita, E. M. Wright, K. Dholakia, Optical binding of two cooled micro-gyroscopes levitated in vacuum. *Optica* **5**, 910–917 (2018).

34. L. Rondin, J. Gieseler, F. Ricci, R. Quidant, C. Dellago, L. Novotny, Direct measurement of Kramers turnover with a levitated nanoparticle. *Nat. Nanotechnol.* **12**, 1130–1133 (2017).

35. J. Millen, T. Deesuwan, P. Barker, J. Anders, Nanoscale temperature measurements using non-equilibrium Brownian dynamics of a levitated nanosphere. *Nat. Nanotechnol.* **9**, 425–429 (2014).

36. J. Gieseler, R. Quidant, C. Dellago, L. Novotny, Dynamic relaxation of a levitated nanoparticle from a non-equilibrium steady state. *Nat. Nanotechnol.* **9**, 358–364 (2014).

37. R. M. Pettit, W. Ge, P. Kumar, D. R. Luntz-Martin, J. T. Schultz, L. P. Neukirch, M. Bhattacharya, A. N. Vamivakas, An optical tweezer phonon laser. *Nat. Photon.* **13**, 402–405 (2019).

38. S. J. Parkin, R. Vogel, M. Persson, M. Funk, V. L. Y. Loke, T. A. Nieminen, N. R. Heckenberg, H. Rubinsztein-Dunlop, Highly birefringent vaterite microspheres: Production, characterization and applications for optical micromanipulation. *Opt. Express* **17**, 21944–21955 (2009).

39. N. Grønbech-Jensen, O. Farago, A simple and effective Verlet-type algorithm for simulating Langevin dynamics. *Mol. Phys.* **111**, 983–991 (2013).

40. S. H. Simpson, P. Zemánek, O. M. Maragò, P. H. Jones, S. Hanna, Optical binding of nanowires. *Nano Lett.* **17**, 3485–3492 (2017).

41. A. Doicu, T. Wreidt, Y. A. Eremin, *Light Scattering by Systems of Particles* (Springer, 2006).

42. S. H. Simpson, S. Hanna, Optical angular momentum transfer by Laguerre-Gaussian beams. *J. Opt. Soc. Am. A* **26**, 625–638 (2009).

43. L. Novotny, B. Hecht, *Principles of Nano-Optics* (Cambridge Univ. Press, 2006).

44. S. H. Simpson, S. Hanna, Stability analysis and thermal motion of optically trapped nanowires. *Nanotechnology* **23**, 205502 (2012).

45. S. H. Simpson, S. Hanna, Holographic optical trapping of microrods and nanowires. *J. Opt. Soc. Am. A* **27**, 1255–1264 (2010).
